# Diabetes-specific enteral nutrition formula in hyperglycemic, mechanically ventilated, critically ill patients: a prospective, open-label, blind-randomized, multicenter study

**DOI:** 10.1186/s13054-015-1108-1

**Published:** 2015-11-09

**Authors:** Alfonso Mesejo, Juan Carlos Montejo-González, Clara Vaquerizo-Alonso, Gabriela Lobo-Tamer, Mercedes Zabarte-Martinez, Jose Ignacio Herrero-Meseguer, Jose Acosta-Escribano, Antonio Blesa-Malpica, Fátima Martinez-Lozano

**Affiliations:** Intensive Care Unit, Hospital Clínico Universitario, Avda Blasco Ibáñez 17, 46010 Valencia, Spain; Intensive Care Unit, Hospital Universitario 12 de Octubre, Avda de Cordoba s/n, 28041 Madrid, Spain; Intensive Care Unit, Hospital Universitario de Fuenlabrada, Camino del Molino 2, 28942 Fuenlabrada, Madrid Spain; Clinic and Dietetics Nutrition Unit, Hospital Virgen de las Nieves, Avda Fuerzas Armadas 2, 18014 Granada, Spain; Intensive Care Unit, Hospital Universitario de Donostia, Paseo Dr. Beguiristain s/n, 20080 Donostia, Spain; Intensive Care Unit, Hospital Universitario de Bellvitge, C/ Feixa Llarga 1, 08907L’Hospitalet de Llobregat, Barcelona, Spain; Intensive Care Unit, Hospital General Universitario, C/ Pintor Baeza s/n, 03005 Alicante, Spain; Intensive Care Unit, Hospital Clínico San Carlos, C/ Prof. Martín Lagos s/n, 28040 Madrid, Spain; Intensive Care Unit, Hospital General Universitario Reina Sofía, Avda Intendente Jorge Palacios 1, 30003 Murcia, Spain

## Abstract

**Introduction:**

Although standard enteral nutrition is universally accepted, the use of disease-specific formulas for hyperglycemic patients is still controversial. This study examines whether a high-protein diabetes-specific formula reduces insulin needs, improves glycemic control and reduces ICU-acquired infection in critically ill, hyperglycemic patients on mechanical ventilation (MV).

**Methods:**

This was a prospective, open-label, randomized (web-based, blinded) study conducted at nine Spanish ICUs. The patient groups established according to the high-protein formula received were: group A, new-generation diabetes-specific formula; group B, standard control formula; group C, control diabetes-specific formula. Inclusion criteria were: expected enteral nutrition ≥5 days, MV, baseline glucose >126 mg/dL on admission or >200 mg/dL in the first 48 h. Exclusion criteria were: APACHE II ≤10, insulin-dependent diabetes, renal or hepatic failure, treatment with corticosteroids, immunosuppressants or lipid-lowering drugs and body mass index ≥40 kg/m^2^. The targeted glucose level was 110–150 mg/dL. Glycemic variability was calculated as the standard deviation, glycemic lability index and coefficient of variation. Acquired infections were recorded using published consensus criteria for critically ill patients. Data analysis was on an intention-to-treat basis.

**Results:**

Over a 2-year period, 157 patients were consecutively enrolled (A 52, B 53 and C 52). Compared with the standard control formula, the new formula gave rise to lower insulin requirement (19.1 ± 13.1 vs. 23.7 ± 40.1 IU/day, *p* <0.05), plasma glucose (138.6 ± 39.1 vs. 146.1 ± 49.9 mg/dL, *p* <0.01) and capillary blood glucose (146.1 ± 45.8 vs. 155.3 ± 63.6 mg/dL, *p* <0.001). Compared with the control diabetes-specific formula, only capillary glucose levels were significantly reduced (146.1 ± 45.8 vs. 150.1 ± 41.9, *p* <0.01). Both specific formulas reduced capillary glucose on ICU day 1 (*p* <0.01), glucose variability in the first week (*p* <0.05), and incidences of ventilator-associated tracheobronchitis (*p* <0.01) or pneumonia (*p* <0.05) compared with the standard formula. No effects of the nutrition formula were produced on hospital stay or mortality.

**Conclusions:**

In these high-risk ICU patients, both diabetes-specific formulas lowered insulin requirements, improved glycemic control and reduced the risk of acquired infections relative to the standard formula. Compared with the control-specific formula, the new-generation formula also improved capillary glycemia.

**Trial registration:**

Clinicaltrials.gov NCT1233726.

**Electronic supplementary material:**

The online version of this article (doi:10.1186/s13054-015-1108-1) contains supplementary material, which is available to authorized users.

## Introduction

Critically ill patients show a stereotype metabolic response to injury that affects carbohydrate metabolism [1, 2], causing hyperglycemia, which is boosted by the actions of counterregulatory hormones [3, 4]. This metabolic response makes the critically ill patient especially susceptible to infection and increases morbidity and mortality [5–7]. The control of blood glucose levels and adequate nutritional support contribute to metabolic improvement and help reduce the risk of infection.

Glycemic variability (GV) has been independently associated with mortality and a higher risk of intensive care unit (ICU)-acquired infection in critically ill patients [8–11]. Accordingly, it has been proposed that this variability could be improved by modifying the composition of the standard enteral nutrition formula given to the patient [12]. In an effort to avoid the detrimental consequences of hypoglycemia [13–15], many ICUs have elevated their targeted lower limits for glucose levels. However, to date no consensus has been reached on the optimal target glucose range [16–19].

Hyperglycemia in the ICU patient can be treated with exogenous insulin [20, 21] and by the enteral administration of diabetes-specific formulas [22]. Although the use of standard enteral nutrition (EN) products is widely accepted, the benefits of disease-specific or modified standard enteral formulas for hospitalized patients with diabetes remain controversial [23, 24]. When used as short- to medium-term treatment, a standard high-carbohydrate/low-fat diet may compromise glycemic control [25, 26]. By modifying carbohydrate composition and adding monounsaturated fatty acids (MUFA) and fiber, studies have shown improved glycemic control compared with a standard diet [27–29]. However, few studies have examined the benefits of diabetes-specific formulas in critically ill patients with hyperglycemia [30].

We hypothesized that in the hyperglycemic, critically ill patient, the use of a diabetes-specific nutrition formula might serve to improve glycemic control and reduce the risk of hospital-acquired infection. The aim of our study was to compare, in mechanically ventilated ICU patients with hyperglycemia, the use of three EN formulas: a standard EN formula, a widely used diabetes-specific formula and a new-generation diabetes-specific formula. This last formula contains high protein levels, MUFA, slowly absorbed carbohydrates, and omega-3 polyunsaturated fatty acids (PUFA), and is enriched with eicosapentaenoic acid (EPA), docosahexaenoic acid (DHA) and fiber.

## Methods

### Study design and data collection

A prospective, multicenter, open-label, blind-randomized, controlled clinical trial was conducted in medical-surgical ICUs in nine teaching hospitals in Spain. The study protocol was approved by each hospital’s review board. A list of ethics committees that approved the study at each institution is provided as Additional file 1. Written informed consent was obtained from participating patients or closest relatives.

The primary endpoint was the amount of insulin required to keep glucose levels in the range 110–150 mg/dL (glycemia target). Secondary endpoints were glycemic control (plasma and capillary blood glucose, glycemic variability), ICU-acquired infection (catheter-related bloodstream infection, primary bloodstream infection, urinary tract infection, ventilator-associated tracheobronchitis incidence rate/1000 ventilator days and ventilator-associated pneumonia incidence rate/1000 ventilator days), days on mechanical ventilation (MV), ICU stay and mortality at 28 days post admission. After ICU discharge, patients were followed for 6 months to record hospital stay, hospital mortality, and 6-month mortality.

### Patient assignment/randomization

The study was performed over the period April 2010 to May 2012. A website was constructed to record data and to randomize patients to each EN formula. Data were collected online using an electronic case report form. For randomization, the website assigned the formulas in a blinded, centralized fashion in blocks of six patients, including at least three blocks stratified by participating center, according to the sequence: study group A, new-generation diabetes-specific high-protein formula (Diaba HP®, Vegenat, Badajoz, Spain); control group B, standard high-protein formula (Isosource Protein Fibra®, Nestlé Health Science, Barcelona, Spain); control group C, widely used diabetes-specific high-protein formula (Glucerna Select®, Abbott Nutrition, Madrid, Spain). Patients were consecutively enrolled at each center by the responsible researcher. When enrolling a patient, the researcher was blind to the randomization sequence. All participants were followed at the ICU for a maximum of 28 days.

### Patient eligibility

Inclusion criteria were: age ≥18 years, ICU stay ≤48 hours upon randomization to EN formula, mechanical ventilation, EN indicated for an expected time ≥5 days. Patients were also required to meet American Diabetes Association criteria for diabetes/hyperglycemia [31] (baseline blood glucose >126 mg/dL after fasting or >200 mg/dL otherwise) in the first 48 h of ICU admission. Exclusion criteria were: contraindication for EN, expected EN <5 days, Acute Physiology and Chronic Health Evaluation II (APACHE II) score ≤10, insulin-dependent diabetes, acute or chronic kidney failure [32], liver failure (total bilirubin ≥3 mg/dL, Child-Pugh B-C), life expectancy ≤48 h, previous cardiac arrest, long-term therapy with corticosteroid, immunosuppressant or lipid-lowering drugs, pregnancy, body mass index (BMI) ≥40 and parenteral nutrition (PN).

### Administration protocol and formula composition

The study patients were started on EN through a nasogastric tube within 48 h of ICU admission. Enteral feeding was continued over 24 h each day and only interrupted for 8 h (from 12 p.m. to 8 a.m.) along with insulin infusion on days 3, 7, 14, 21 and 28 to obtain samples for complete blood tests. Patients were managed by the responsible researchers at each center. Gastric residual volume (GRV) was measured every 6 h for the first 2 days and every 24 h thereafter until EN was stopped. All patients received 10 mg metoclopramide every 8 h only for the first 3 days.

Prescribed caloric intake was 25 kcal/kg/day. Nutritional requirements were calculated by the responsible researchers. It was ensured that patients received their calculated requirements in the first 48 h of EN. Non-nutritional caloric constituents like glucose or propofol were included in caloric balance calculations. Insulin was administered in saline solution at a 1:1 ratio by continuous infusion using a 50-mL syringe pump. The nursing staff adjusted the dose guided by capillary blood glucose levels using a sliding-scale algorithm and a point-of-care glucose meter following a consensus protocol for the nine participating centers (see Table S1 in Additional file 2).

Patients were randomized to receive one of three different nutrition formulas (Table 1). The two diabetes-specific formulas contain greater percentages of fat while the standard formula has a greater carbohydrate content and less fiber. The carbohydrates included in each formula are: low-dextrose-equivalent maltodextrin and type IV-resistant maltodextrin in the study formula; modified maltodextrin, fructose and maltitol in the control diabetes-specific formula; and maltodextrin and saccharose in the standard high-protein formula. The two specific formulas contain greater proportions of MUFA and the study diet also has higher ω-3, EPA and DHA contents. Due to their different caloric densities, formula volumes were calculated on the website to ensure patients received similar caloric and nitrogenous support.

**Table 1:** Composition of enteral formulas per 100 kcal and per 100 ml

Nutritional information	DIABA HP®	ISOSOURCE PROTEIN FIBRA®	GLUCERNA SELECT®
Caloric density (kcal : ml)	1 : 1	1.4 : 1	1 : 1
Energy/volume	Per 100 kcal / per 100 ml	Per 100 kcal / per 100 ml	Per 100 kcal / per 100 ml
Protein (g)	5.7	5.00 / 7.00	5.00
	Casein 50 %	Casein 100 %	Casein 80 %
	Whey P + GMP 25 %		Soy P 20 %
	Vegetable P 25 %		
Carbohydrates (g)	8.2	10.93/15.3	7.46
	Modified maltodextrin (low dextrose equivalent and type IV resistant)	Standard maltodextrin	Modified maltodextrin
		Sucrose	Fructose
			Maltitol
Fat (g)	4.4	3.79 / 5.3	5.44
Saturated (g)	1.1	1.00 / 1.4	0.45
Monounsaturated (g)	2.2	1.43 / 2	3.58
Polyunsaturated (g)	1.00	1.37 / 1.92	1.14
EPA + DHA (mg)	67.6	-	-
Fiber (g)	1.8	1.07 / 1.5	1.44
Soluble	1.4	0.5 / 0.7	0.21
Insoluble	0.4	0.57 / 0.8	1.23
Minerals			
Calcium (mg)	80	53.5 / 75	70
Phosphate (mg)	75	53.5 / 75	65
Potassium (mg)	200	96.4 / 135	130
Sodium (mg)	70	60.7 / 85	94
Chlorine (mg)	70	107.1 / 150	125
Iron (mg)	1.1	0.79 / 1.1	1.3
Zinc (mg)	1.1	0.79 / 1.1	1.2
Copper (μg)	113	0.10 / 0.14	140
Iodine (μg)	10	8.57 / 12	11
Selenium (μg)	4.8	3.29 / 4.6	5
Magnesium (mg)	16	15.71 / 22	21
Manganese (mg)	0.15	0.16 / 0.22	0.35
Fluoride (mg)	0.12	0.11 / 0.16	0
Molybdenum (μg)	4.4	4.14 / 5.8	10
Chromium (μg)	2	2.86 / 4	8.5
Vitamins			
A (μg)	85	57.14 / 80	58
D (μg)	1.3	0.71 / 1	0.93
E (mg)	1.5	1.14 / 1.6	1.9
C (mg)	9.1	3.57 / 5	11
B_1_ (mg)	0.22	0.09 / 0.12	0.15
B_2_ (mg)	0.22	0.11 / 0.16	0.18
B_3_ (mg)	1.5	1.29 / 1.8	1.7
B_6_ (mg)	0.22	0.11 / 0.16	0.21
B_9_ (mg)	27	14.29 / 20	25
B_12_ (μg)	0.32	0.26 / 0.36	0.3
Biotin (μg)	4	7.29 / 10.2	4
Pantothenic A. (mg)	0.8	0.31 / 0.44	0.75
K (μg)	5.2	5.71 / 8	10
Choline (μg)	37	-	43
Osmolarity (mOsm/L)	345	399	378

*P*protein, *GMP* glycomacropeptide, *EPA* eicosapentaenoic acid, *DHA* docosahexaenoic acid, *A* acid

### Variables and controls

Baseline data were collected before starting EN (Table 2). Both the Sequential Organ Failure Assessment (SOFA) and Homeostasis Model Assessment (HOMA2) [33] were repeated on days 3, 7, 14, 21 and 28 after ICU admission.

**Table 2:** Demographics and baseline clinical characteristics of the study population

VARIABLE	GROUP A	GROUP B	GROUP C
	(n = 52)	(n = 53)	(n = 52)
Age (years)	57 (43–70)	60 (45–71)	58 (46–68)
Male (N and %)	37 (71.1 %)	43 (81.1 %)	39 (75 %)
BMI (kg/m^2^)	26 (24–29)	26 (24–28)	26 (24–27)
APACHE II	17 (14–23)	19 (15–22)	19 (16–23)
SOFA score	8 (6–14)	7 (5–12)	7 (4–12)
Calorie requirements (kcal/day)	1690.4 (384.9)	1707.1 (275.4)	1728.2 (309.2)
Start of EN (hours)	23.4(18.1-27.2)	25.1 (19.6-26.2)	26.7 (21.3-28)
Noninsulin-dependent diabetes (N and %)	11 (21.1 %)	7 (13.2 %)	12 (23 %)
HbA1c (%)	6.34 (1.38)	5.76 (0.95)	6.07 (1.39)
Capillary glucose level (mg/dL)^a^	148.8 (38.8)	149.9 (38.9)	151.5 (39.23)
Plasma glucose level (mg/dL)^a^	152.1 (54.6)	147.01 (55.93)	152.1 (57.12)
Insulinemia (μU/mL)^a^	21.59 (36.06)	17.39 (23.77)	19.15 (20.5)
C-peptide (ng/mL)^a^	4.61 (3.24)	4.49 (2.92)	4.27 (3.67)
HOMA2 IR	2.94 (4.19)	2.48 (2.76)	3.38 (2.37)
HOMA2 %B (β-cell)	102.6 (57.81)	110.5 (74.34)	96.55 (85.09)
HOMA2 %S	79.12 (75.2)	69.6 (48.7)	68.09 (66.9)
Infection (any source) (N and %)	13 (25 %)	15 (28.3 %)	8 (15.3 %)
Diagnostic group (N and %)			
Medical	32 (61.53 %)	36 (67.93 %)	30 (57.69 %)
Trauma	13 (25 %)	9 (16.98 %)	15 (28.85 %)
Surgery	7 (13.47 %)	8 (15.09 %)	7 (13.46 %)

Values are provided as mean (SD), median (Q_1_-Q_3_) and percentages (%)

*N* number, *BMI* body mass index, *APACHE II*, Acute Physiology and Chronic Health Evaluation II, *SOFA* Sequential Organ Failure Assessment, *EN* enteral nutrition, *HbA1c* glycated hemoglobin, *HOMA2* Homeostasis Model Assessment, *IR* insulin resistance, *%S* insulin sensitivity, *%B* beta cell function^a^Measured after fasting and without insulin infusion for at least 8 hours

Variables were monitored for a maximum of 28 days. The variables MV days, EN days and ICU stay were recorded when the patient was discharged from the ICU. Hospital stay and mortality were recorded at 28 days and 6 months.

Complete blood tests were conducted on a peripheral blood sample on ICU admission. Blood samples for similar tests on days 3, 7, 14, 21 and 28 post admission were obtained at 8 a.m., 8 hours after stopping EN and insulin infusion.

The glycemic target was set at 110–150 mg/dL. For the present purposes, severe hypoglycemia was defined as a blood glucose level less than 50 mg/dL and moderate hypoglycemia as a level of 50–80 mg/dL. Insulin infusion was started when glucose levels reached ≥150 mg/dL. To monitor daily plasma glucose levels, venous blood was withdrawn from each patient at 8 a.m.; capillary blood glucose levels were monitored every 1–4 h using a Glucometer Elite XL from Bayer®, Barcelona, Spain. Plasma glucose levels were measured by the hexokinase method at each center’s main laboratory.

In each patient, we recorded mean and standard deviation (SD) plasma glucose levels. Daily mean (Glu_M_) and standard deviation (Glu_SD_) capillary blood glucose levels were obtained during their ICU stay. Insulin (in international units) administered to each patient per day was also recorded.

The following glycemia control indices were recorded for each patient [8]: plasma glucose level on admission; mean and SD of capillary blood glucose levels recorded on ICU day 1; peak glucose during ICU stay; and GV during the first 7 days and during the 28 days of the study. Glycemic variability was measured as the mean SD of daily capillary glucose levels, the coefficient of variation (CV) according to the equation Glu_CV_ = Glu_SD_ × 100/Glu_M_, and the glycemic lability index (GLI). The GLI was calculated using the Ryan equation [34] modified for critically ill patients and corrected for the number of blood extractions as follows:

$$ GLI\left\{\frac{{\left(\frac{mmol}{l}\right)}^2}{h}\times da{y}^{-1}\right\}=\sum_{n=1}^N\frac{{\left(Glu{c}_n-Glu{c}_{n+1}\right)}^2}{\left(N-1\right)\times \left({h}_{n+1}-{h}_n\right)} $$

where Gluc_n_ is the nth reading for the patient at time n measured in mmol/L, N is the total number of readings performed that day, and h_n_ is the time in hours of the nth reading at time n.

Patients were examined daily to detect the presence of gastrointestinal complications. The following gastrointestinal complications were defined according to criteria published by our group [35, 36]:

a) Abdominal distention: tympany and/or absence of bowel sounds; b) high GRV: recovered gastric volume equal to or greater than 500 mL; c) vomiting: enteral formula ejected through the mouth; d) diet regurgitation: enteral formula found in oral or nasal cavities with or without exteriorization; e) diarrhea: five or more liquid stools in a 24-h period or an estimated stool volume equal or greater than 2000 mL/day.

If a complication required withholding the diet for >48 h or starting PN, the case was closed but included in the intent-to-treat analysis.

The volume ratio (VR) was estimated as a measure of the efficacy of daily nutritional administration and calculated as follows: VR (%) = (volume administered/volume prescribed) × 100. Also calculated were calorie and nutrient (carbohydrates, fats and proteins) intakes per patient/day based on daily infused volumes and formula composition.

ICU-acquired infections were defined according to criteria for critically ill patients emerging from consensus conferences [37, 38]. An infection was recorded as acquired if starting 48 h after ICU admission. Biological samples for microbiological cultures were obtained when there was clinical suspicion of infection or at least once a week. The following variables were recorded: number of infected patients, infection rate/100 days of ICU stay, tracheobronchitis/1000 days of MV, ventilator-associated pneumonia/1000 days of MV, bacteremia and urinary tract infection.

### Power calculations and data treatment

Based on the results of our previous study [30], an insulin requirement reduction of at least 20 % was considered clinically meaningful. Assuming an *α* risk of 0.05 and a *β* risk of 0.20 (power = 80 %), the required number of patients was estimated at 159. Each case was recorded in the electronic logbook by the responsible researcher, sent to the principal investigators for verification, and entered into a central database. Each case was checked to ensure that the patient met the inclusion/exclusion criteria, the study protocol had been adequately followed and all the required data had been provided. Discrepancies and transcription errors were discussed among the researchers and clarified by the principal investigators.

An intent-to-treat analysis was performed. Qualitative variables are expressed as absolute and relative frequencies and continuous variables as means (±SD) or as medians (quartile 1; quartile 3). Qualitative variables were compared using the chi-squared test or Fisher’s exact test. Continuous data were assessed for normality using the Kolmogorov-Smirnov test. The nonparametric Kruskal-Wallis and Mann-Whitney *U* tests were used when the data did not fit the assumptions of normality. If the dependent variable under examination was quantitative, we statistically assessed the comparability of the three treatment groups by one-way analysis of variance (ANOVA) and the corresponding multiple comparison of means test (Tukey’s HSD, Bonferroni). For the main outcome measures, a repeated measures generalized linear model (GLM) was used when considering measurements taken at different time points in a single patient.

For incidence rates, we used inference methods on variables recorded in two populations, obtaining a *p* value for the chi-squared test. The relationship between glucose variability and acquired infection complication rate adjusted for time was calculated using the linear correlation coefficient (Pearson’s *r* test).

All statistical tests were performed using the SAS statistical analysis system package (version 9.3, SAS Institute, Cary, NC, USA) SPSS (version 21, IBM Corp., Armonk, NY, USA) and the epidemiological analysis program EPIDAT for tabulated data (version 3.1, EpiData Association, Odense, Denmark). Significance was set at *p* <0.05.

## Results

### Baseline characteristics

**Fig. 1:**
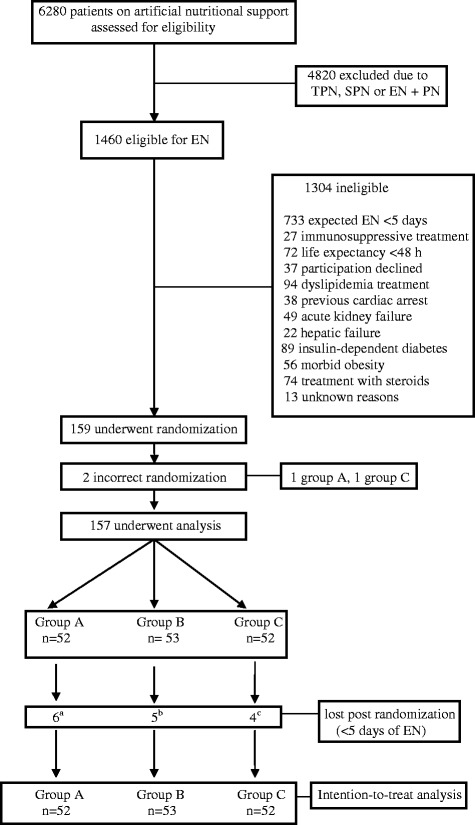
Enrollment, randomization and follow-up of the study participants. *TPN* total parenteral nutrition, *SPN* supplementary parenteral nutrition, *EN* enteral nutrition. **a** Three deaths, one withdrawal by patient, one withdrawal by doctor, one change of diet. **b** Two deaths, one withdrawal by doctor, one change of diet, one infusion interrupted >48 h. **c**Two deaths, one change of diet, one infusion interrupted >48 hours

A total of 159 consecutive ICU patients fulfilling the inclusion criteria were initially recruited. Two patients were excluded because of incorrect randomization (one group A, one group C) leaving a study population of 157 patients (52 group A, 53 group B, 52 group C) (Fig. 1). Fifteen patients did not complete the minimum of 5 days of treatment, but all 157 were included in the intent-to-treat analysis. The three patient groups were homogeneous in terms of demographic and baseline clinical characteristics (Table 2).

Nutrition onset was targeted for within 24 h of ICU admission. Median values for EN onset after ICU admission in hours were 23.4 (18.1–27.2), 25.1 (19.6–26.2) and 26.7 (21.3–28) for groups A, B and C, respectively (Table 2).

The calorie and protein intake values provided in Table 3 indicate isocaloric and isonitrogenous intakes across the three groups, with no differences in effective volume. The mean volume ratio was approximately 80 % of the prescribed ratio. Despite differences in fiber, carbohydrate and lipid intake arising from the different formula compositions, total intakes were within recommended limits. Propofol 2 % was administered in the first 7 days of ICU stay in six group A, seven group B, and five group C patients. The maximum dose received by these patients was 2.2 g/d and the kcal provided by this agent was included in the calorie intake calculations. The intravenous administration of glucose 5 % in three group A, two group B and two group C patients some time during their ICU stay was also taken into account as a source of calories.

**Table 3:** Nutrients received

	GROUP A	GROUP B	GROUP C	*p* value
	(n = 52)	(n = 53)	(n = 52)	
Volume ratio (%)	79.8 (13.4)	81.1 (17)	82.4 (15)	0.71
Total calories received (kcal/day)	1349 (323)	1385 (454)	1423 (383)	0.17
(kcal/kg/day)	22.2 (4.1)	21.7 (4.8)	21.3 (3.8)	
Protein (g/day)	76.8 (18.4)	69.2 (23.2)	71.1 (19.1)	0.11
(g/kg/day)	1.26 (0.32)	1.21 (0.27)	1.23 (0.18)	
Carbohydrates (g/day)	110.6 (25.8)	151.3 (50.7)^*a^	106.1 (28.6)	<0.001^*^
(g/kg/day)	1.6 (0.18)	2.1 (0.26)	1.5 (0.19)	
Fat (g/day)	59.3 (16.4)	52.4 (17.5)^*b^	77.4 (20.8)^*c^	<0.01^*b^
(g/kg/day)	0.8 (0.12)	0.71 (0.17)	0.96 (0.22)	<0.001^*c^
Fiber (g/day)	19.1 (5.8)	13.4 (4.9)^a*^	15.7 (5.5)^*d^	<0.01^*d^
(g/kg/day)	0.26 (0.09)	0.16 (0.05)	0.2 (0.06)	<0.001^*a^

Values are provided as means (SD). Asterisks indicate statistical significance

^a^Difference group B vs. groups A and C

^b^Difference group A vs. group B

^c^Difference group C vs. groups A and B

^d^Difference group A vs. group C

### Glycemic and metabolic control

The variables recorded related to glycemic control are provided in Table 4. These data indicate that patients in group A needed less insulin (*p* <0.05) and showed lower plasma and capillary blood glucose levels than those in group B (*p* <0.01 and *p* <0.001 respectively). No differences were detected in insulin dose between groups A and C; however, in the group A patients, capillary blood glucose was significantly lower (*p* <0.01). Both diabetes formulas achieved significant improvements in mean capillary glycemia (MCG) on the first day of ICU stay compared with group B (*p* <0.01). No differences emerged among the three groups in peak glycemia, number of capillary glycemia measurements and number of measurements made per patient and day.

**Table 4:** Variables related to glycemic control

	GROUP A	GROUP B	GROUP C	*p* value
	(n = 52)	(n = 53)	(n = 52)	
Administered insulin (IU/day)	19.1 (13.1)	23.7 (40.1)^*a^	20.3 (30.1)	<0.05^*^
Plasma glucose level (mg/dL)	138.6 (39.1)	146.1 (49.9)^*a^	143.9 (45.9)	<0.01^*^
Capillary glucose level (mg/dL)	146.1 (45.8)	155.3 (63.6)^*a^	150.1 (41.9)^*b,c^	<0.001^*a^
				<0.01^*b,c^
Mean capillary glycemia on ICU day 1 (mg/dL)	147.5 (40.2)	160 (55.5)^*d^	145.6 (46.6)	<0.01^*^
Peak glucose level (mg/dL)	181.3 (52)	193.6 (74.6)	191.3 (65.8)	0.68
Number of capillary glycemia measurements	3605	3523	3557	0.57
Number of measurements per patient/day	5.7 (3.4)	5.81 (3.2)	5.46 (2.9)	0.56
Percentage of controls on 80–150 mg/dL	59 %	57.6 %	59.59 %	0.82
Hypoglycemia (50–80 mg/dL)	53 (1.48 %)	127 (3.63 %)^*d^	44 (1.25 %)	<0.05^*^
Hypoglycemia (<50 mg/dL)	-	4 (0.11 %)	1 (0.02 %)	0.32
Capillary glucose SD	45.83	63.67^*d^	41.98	<0.01^*^
Glycemic lability index (ICU days 1–28)	0.58 (0.2–1.4)	0.71 (0.3–1.9)^*d^	0.44 (0.2–1.2)	<0.05^*^
Glycemic lability index (ICU days 1–7)	0.27 (0.1–0.7)	0.6 (0.2–1.2)^*d^	0.27 (0.2–0.8)	<0.05^*^
Glycemic variability SD (mg/dL) (ICU days 1–28)	33.6 (18.4)	49.1 (21.5)^*a^	41.1 (9.3)	<0.01^*^
Glycemic variability SD (mg/dL) (ICU days 1–7)	43.2 (4.9)	68.5 (13.5)^*d^	42.5 (2.7)	<0.01^*^
Glycemic CV (%) (ICU days 1–28)	27.9 (5.8)	32.4 (11.2)	27.8 (5.2)	0.13
Glycemic CV (%) (ICU days 1–7)	28.3 (1.9)	42.6 (8.2)^*d^	28.4 (2.1)	<0.001^*^

Values are provided as mean (SD) and percentages (%). Asterisks indicate statistical significance

*IU*, international units, *ICU* intensive care unit, *SD*, standard deviation, *CV* coefficient of variation

^a^Difference group A vs. group B

^b^Difference group B vs. group C

^c^Difference group A vs. group C

^d^Difference group B vs. groups A and C

The rate of severe hypoglycemia episodes (≤50 mg/dL) was <1 %, with no clinical repercussions. Group B showed the greater frequency of moderate hypoglycemia (50–80 mg/dL).

No significant differences in GV were observed between patients receiving the study diabetes-specific formula and those given the control diabetes-specific formula. Both specific formulas achieved a reduction in glycemic variability relative to the standard formula in the first week of ICU stay (GLI *p* <0.05, SD *p* <0.01, CV *p* <0.001). Over the 28 days of follow- up, reductions were also produced in GLI (*p* <0.05) and SD (*p* <0.01).

Plasma levels of cholesterol, triglycerides, albumin and prealbumin were similar across the three treatment groups.

### Infection control

Infectious complications recorded in the study participants are provided in Table 5. Numbers of infected patients and incidences of any infection per 100 days of treatment were lower in the study group, but not significantly (*p* = 0.54 and *p* = 0.51 respectively). Catheter-associated bloodstream infection, primary bloodstream infection and urinary tract infection did not vary across the groups. Rates of ventilator-associated tracheobronchitis and pneumonia per 1000 days of MV were significantly lower for the study (*p* <0.01 and *p* <0.05 respectively) and control (*p* <0.01) diabetes formulas than the standard formula. No significant differences in infectious complications were produced in patients randomized to receive the study or control diabetes formulas.

**Table 5:** Acquired infectious complications

	GROUP A	GROUP B	GROUP C	*p* value
	(n = 52)	(n = 53)	(n = 52)	
Number of infected patients	18/52 (34.6 %)	23/53 (43.4 %)	23/52 (44.2 %)	0.54
Odds ratio	1.4 (0.6–3.1)^a^		1.03 (0.4–2.2)^c^	
	1.4 (0.6–3.3)^b^			
Infectious complication incidence rate / % treatment days	22/546 (4.03)	24/470 (5.11)	24/505 (4.75)	0.51
Catheter-related bloodstream infection	1/52 (1.92 %)	1/53 (1.89 %)	2/52 (3.85 %)	0.53
Primary bloodstream infection	3/52 (5.77 %)	1/53 (1.89 %)	3/52 (5.77 %)	0.25
UTI	1/52 (1.92 %)	1/53 (1.89 %)	1/52 (1.92 %)	1.00
Tracheobronchitis incidence rate / 1000 ventilator days	7/460 (15.2)^*a^	10/392 (25.5)	7/424 (16.5)^*c^	<0.01^*^
VAP incidence rate / 1000 ventilator days	8/460 (17.3)^*a^	10/392 (25.5)	6/424 (14.1)^*c^	<0.05^*a^
				< 0.01^*c^

Values are provided as percentages (%) and incidence rate. Asterisks indicate statistical significance

*UTI* urinary tract infection, *VAP* ventilator-associated pneumonia

^a^Difference group A vs. group B

^b^Difference group A vs. group C

^c^Difference group B vs. group C

We also examined the relationship between glycemic variability and acquired infections (Table 6). Thus, the incidence of tracheobronchitis per 1000 days of MV showed high significant correlation with glycemic CV for the first week of ICU stay (*r* = 0.997; *p* = 0.04). This variable also showed high correlation, albeit without significance, with GLI recorded over 28 days (*r* = 0.989; *p* = 0.09) and 1 week (*r* = 0.997; *p* = 0.05) of follow-up, and with SD for 1 week (*r* = 0.995; *p* = 0.06) and CV for 28 days (*r* = 0.995; *p* = 0.06) of follow-up. The incidence of ventilator-associated pneumonia / 1000 days of MV only showed high correlation but without significance with the MCG recorded in the first ICU stay (*r* = 0.988; *p* = 0.09).

**Table 6:** Relationship between glycemic control and infectious complications determined through Pearson linear correlation (r)

	Pneumonia / 1000 ventilator days	*p* value	Tracheobronchitis / 1000 ventilator days	*p* value
Mean plasma glucose level	*r* = 0.512	0.65	*r* = 0.778	0.43
Mean capillary glucose level	*r* = 0.749	0.46	*r* = 0.933	0.23
Administered insulin	*r* = 0.863	0.33	*r* = 0.985	0.11
Mean capillary glucose on day 1	*r* = 0.988	0.09	*r* = 0.980	0.12
Variability (GLI) days 1–28	*r* = 0.978	0.13	*r* = 0.989	0.09
Variability (GLI) days 1–7	*r* = 0.962	0.17	*r* = 0.997	0.05
Variability (SD) days 1–28	*r* = 0.713	0.49	*r* = 0.912	0.26
Variability (SD) days 1–7	*r* = 0.969	0.16	*r* = 0.995	0.06
Coefficient of glycemic variation days 1–28	*r* = 0.967	0.16	*r* = 0.995	0.06
Coefficient of glycemic variation days 1–7	*r* = 0.961	0.17	*r* = 0.997	0.04

*GLI* glycemic lability index, *SD* standard deviation

### Other nutritional and clinical outcomes

Diet tolerance was good in all three treatment groups. Only in 11 patients (7 %) did EN have to be interrupted for more than 48 h due to gastrointestinal complications. The complications observed were abdominal distension in one patient (group B), diarrhea in six patients (two group A, one group B and three group C) and high GRV in four patients (one group A, two group B and one group C). The remaining clinical outcomes are shown in Table 7. No significant differences among groups were produced in the number of days on insulin treatment or on enteral nutrition, number of days on MV, ICU and hospital stay, and mortality at 28 days and at 6 months. Figure S1 in Additional file 3 provides the numbers of patients remaining in the study during the 28-day course of follow-up.

**Table 7:** Other clinical outcomes

	GROUP A	GROUP B	GROUP C	*p* value
	(n = 52)	(n = 53)	(n = 52)	
Days on insulin treatment	7.1 (2.8)	7.9 (3.2)	7.7 (3.9)	0.41
Days on enteral nutrition	10.5 (7.1)	8.8 (6.7)	9.7 (7.4)	0.39
Days on mechanical ventilation	7 (4–13)	6 (2–11)	6 (3–12)	0.53
ICU stay (days)	13 (9–20)	12 (7–21)	11.5 (7.5–18)	0.42
Hospital stay (days)	27 (18–50)	25 (17–51)	30.5 (14–46.5)	0.98
28-day mortality (N and %)	11 (21.1 %)	10 (18.87)	13 (25 %)	0.73
6-month mortality (N and %)	16 (30.7 %)	20 (37.74)	18 (34.62)	0.71
Δ SOFA	−0.37	−0.28	−0.33	0.42
Δ Triglycerides	+0.02	−0.21	+0.12	0.18
Δ HOMA2 IR	−0.17	−0.01	−0.22	0.76
Δ HOMA2 % S	+0.17	+0.09	+0.12	0.89
Δ HOMA2 β-cell	+0.21	+0.12	+0.09	0.81

Values are provided as mean (SD), median (Q_1_-Q_3_) and percentages (%)

*ICU* intensive care unit, *N* number, *Δ* delta baseline-ICU discharge, *SOFA* Sequential Organ Failure Assessment, *HOMA2*, Homeostasis Model Assessment, *IR* insulin resistance, *%S* insulin sensitivity, *%B* beta cell function

## Discussion

This study shows that a diabetes-specific high-protein EN formula containing MUFA, slowly absorbed carbohydrates and omega-3 PUFA enriched with EPA, DHA and fiber gives rise to better glycemic homeostasis than a standard high-protein formula and may also reduce the risk of acquired infections in ICU patients.

When comparing the diabetes-specific study formula (A) with a widely used diabetes-specific formula of different composition (C), the only significant difference found was a lower mean capillary blood glucose level in patients given the study formula.

The similar 28-day and 6-month mortality rates recorded for the three treatment groups could be related to an insufficient sample size for comparing these endpoints. No effects of the different formulas were produced on insulin resistance or sensitivity determined using the HOMA2 calculator.

### Glycemic control

Continuous insulin infusion protocols help to maintain plasma glucose levels within recommended ranges [14, 15, 18–20]. However, consistent with our findings, numerous studies have shown that insulin requirements and plasma and capillary blood glucose levels can be reduced using a diabetes-specific EN formula as an adjuvant to insulin [22, 25, 27, 29, 30] (Table 4). It therefore seems that the composition of these formulas, both in quantitative terms (smaller percentages of carbohydrates and higher fat percentages) and qualitative terms (including modified carbohydrates and MUFA, EPA and DHA), helps reduce insulin requirements, and this also reduces the risk of hypoglycemia. No prior studies in critical patients have compared enteral feeding formulas with similar protein and fat contents but different carbohydrate sources and levels such that we cannot attribute the better blood glucose control observed here to the carbohydrate supply provided by the formula. Notwithstanding, recent clinical practice guidelines [23, 24] do not recommend the use of this type of formula and indicate a need for further studies.

For some years, GV has been associated with outcome in the critically ill patient. Thus, in a cohort of 7049 critical patients, Egi et al. [8] observed a greater GV in patients who died than in survivors. Similarly, Hermanides et al. [9], in 5728 critical patients, found a significant relationship between GV, measured as mean absolute glucose, and the likelihood of ICU death. In addition, Krinsley et al. [10], in an international, multicenter cohort study conducted in 44,964 patients, noted that greater GV was independently associated with increased mortality in critical patients with no prior diabetes.

These studies on GV, however, failed to address the nutritional support or, in many cases, the amount of insulin administered to the patients. Thus, it is worth asking what influence these diabetes-specific formulas could have in controlling both hyperglycemia and GV.

Alish et al. [12] reported lower GV based on the mean amplitude of glycemic excursions (MAGE) in noncritically ill patients with diabetes who were administered a diabetes-specific formula than in those administered a standard formula. These results are in agreement with those of our study (Table 4) indicating similarly lower glucose variability for the diabetes-specific study and control formulas versus the standard formula, both in the first week of ICU stay and for 28 days of follow-up. In the first 7 days of ICU stay, stress hypermetabolism is at its maximum and its adequate control can impact short-term outcomes in critically ill patients. In our patients, greatest differences among treatment groups were detected in MCG on the first ICU day and in GV and CV during the first ICU week. Given the important role of GV in critically ill patient outcomes, we recommend the use of such specific formulas in hyperglycemic ICU patients, at least during the first week of ICU stay.

### Infection control

A relationship between hyperglycemia and infection has been established in several populations of critically ill patients [39–41]. In a meta-analysis of data from 29 randomized, controlled trials including 8432 patients, fewer septic complications were observed in patients subjected to strict glycemic control [18]. Similarly, Van den Berghe et al. [16] noticed fewer infections in surgical patients in whom a tight glycemic control protocol had been followed. However, these findings were not confirmed in a subsequent study in medical patients [42], in the NICE-SUGAR study [14] or in a subsequent meta-analysis [15].

In our study, fewer ICU-acquired infections were observed in patients on both diabetes-specific high-protein formulas than those given the standard high-protein formula (Table 5). The differences in insulin needs and in plasma and capillary glycemia between these groups could only partially explain this reduction. We also analyzed (Table 6) the relationship between glycemic control and any significant infections identified in the group comparisons. Thus, as stated in the results section, high significant correlation (*r* >0.9) with tracheobronchitis episodes per 1000 days of MV was detected for glycemic CV in the first week of ICU stay and a trend toward significance was noted for the other glycemia variability parameters. This correlation with infection was not observed for the other variables such as the amount of insulin administered or capillary blood and plasma glucose levels.

As far as we know, no similar data exist in the medical literature linking rates of infectious complications to the administration of diabetes-specific formulas in critically ill patients. In a retrospective study of 2782 patients admitted to a medical-surgical ICU, Donati et al. [11] were the first to report close correlation between glycemic variability (GLI, SD, CV and MAGE) and acquired infections in diabetic and nondiabetic patients, with higher discriminative values observed for GLI and CV. Our findings also point to this correlation though since it was not a primary endpoint this correlation needs to be confirmed.

Many retrospective studies have revealed a relationship between high glycemic variability and poor outcomes in critically ill patients. To date, however, there are no sufficiently established standardized methods to control such variability. We could nevertheless speculate that, by reducing insulin requirements and plasma and capillary glucose levels, the use of specific formulas for diabetes would be useful for this purpose. Further prospective, randomized studies in large patient series are warranted to confirm this relationship and its potential therapeutic consequences.

### Strengths and limitations

As strengths of this study, we should mention the idea that glycemic control can be modified using specific enteral nutrients in the critically ill patient; also the external validity of the methodology (prospective, blind-randomized, multicenter) and the generalizability of results. Its main limitations are that it was an open-label study, so some of its results (especially effects on infectious complications) need to be considered with caution. The use of capillary samples for blood glucose instead of arterial samples and use of the point-of-care glucose meter could be considered a further limitation. Consensus recommendations published in 2013 [43] highlight the need to obtain arterial rather than capillary blood samples. This was selected as the standardized method by the researchers at the nine hospitals given it was logistically nonviable to collect arterial blood samples at the frequency necessary at two of the centers. However, regardless of the greater or lesser reliability of absolute glycemia values, the use of capillary samples should not affect the comparisons made between the three groups of patients. As a final limitation, the sample size precluded reaching conclusions on outcome measures (such as mortality) other than those related to glycemic or infection control.

Although the components of a diabetic-specific formula are important, no marked differences were observed in the impacts of the two such formulas examined here. Thus, to confirm the present findings and further examine EN effects on glycemic variability and mortality, we recommend comparing the latest-generation diabetes-specific formula with a standard formula in a multicenter, double-blind trial in a large patient sample.

## Conclusions

The use of a high-protein diabetes-specific EN formula in hyperglycemic critically ill patients on mechanical ventilation leads to lower insulin requirements, reduces plasma and capillary blood glucose levels and glycemic variability, and could also reduce the risks of ICU-acquired ventilator-associated pneumonia and tracheobronchitis when compared with that of a standard high-protein formula. Similar findings were obtained for the use of another diabetes-specific formula of different composition.

Our results highlight the need for double-blind studies with glycemic variability as the primary endpoint in large critically ill patient populations to address the impacts of these enteral nutrition formulas on outcomes such as acquired infections, hospital stay and short- and medium-term mortality.

## Key messages

- Few studies have explored the possible benefits of diabetes-specific EN formulas in terms of controlling hyperglycemia, glucose variability and acquired infectious complications.
- In our study, compared with a high-protein standard EN formula, a high-protein diabetes-specific formula reduced insulin requirements, glucose variability, and plasma and capillary glycemia in mechanically ventilated, hyperglycemic ICU patients.
- Our results also point to beneficial impacts of such a formula on ICU-acquired infections such as ventilator-associated tracheobronchitis and pneumonia. However, further efforts in the form of larger clinical trials are required to assess the capacity of these formulas to reduce the risk of acquired infections in these patients.

## Additional files

Additional file 1:
**Ethics committees that approved the study at each institution.** (DOC 24 kb) Additional file 2: Table S1. Insulin therapy protocol. (DOCX 19 kb) Additional file 3: Figure S1. Patient numbers per treatment group during the course of follow-up. (DOCX 12 kb)

## Abbreviations

ANOVA: analysis of variance
APACHE: Acute Physiology and Chronic Health Evaluation
BMI: body mass index
CV: coefficient of variation
DHA: docosahexaenoic acid
EN: enteral nutrition
EPA: eicosapentaenoic acid
GLI: glycemic lability index
GLM: generalized linear model
GRV: gastric residual volume
GV: glucose variability
HOMA: Homeostasis Model Assessment
ICU: intensive care unit
MAGE: mean amplitude of glycemic excursions
MCG: mean capillary glycemia
MUFA: monounsaturated fatty acid
MV: mechanical ventilation
PN: parenteral nutrition
PUFA: polyunsaturated fatty acid
SD: standard deviation
SOFA: Sequential Organ Failure Assessment
VR: volume ratio
