# Supplementary material for: Diabetes-specific enteral nutrition formula in hyperglycemic, mechanically ventilated, critically ill patients: a prospective, open-label, blind-randomized, multicenter study
Source: Crit Care. 2015 Nov 9;19:390. doi: 10.1186/s13054-015-1108-1 (PMC4638090; doi:10.1186/s13054-015-1108-1)
Supplement: Additional file 1: — Ethics committees that approved the study at each institution. (DOC 24 kb) [file 13054_2015_1108_MOESM1_ESM.doc]

Supplementary material-3

Ethics Committees that approved the study at each institution.

1. Comité Etico de Investigación Clínica. (*Ethics in Clinical Investigation Committee*) Hospital Clínico Universitario, Valencia, Spain. (REF:39/09)
2. Comité Etico de Investigación Clínica. (*Ethics in Clinical Investigation Committee*) Hospital Universitario 12 de Octubre, Madrid, Spain. (REF: 09/227)
3. Comité Etico de Investigación Clínica. (*Ethics in Clinical Investigation Committee*) Hospital Universitario de Fuenlabrada, Fuenlabrada, Madrid, Spain (REF: IR2009)
4. Comité Etico de Investigación Clínica. (*Ethics in Clinical Investigation Committee*) Hospital Virgen de las Nieves, Granada, Spain. (REF: 18/18/2009)
5. Comité Etico de Investigación Clínica de Euskadi. (*Ethics in Clinical Investigation Committee*) Vitoria-Gasteizt. Spain. (REF:02/02/2010)
6. Comité Etico de Investigación Clínica. (*Ethics in Clinical Investigation Committee*) Hospital Universitario de Bellvitge, L’Hospitalet de Llobregat, Barcelona, Spain. (REF: PR239/09)
7. Comité Etico de Investigación Clínica. (*Ethics in Clinical Investigation Committee*) Hospital General Universitario, Alicante, Spain. (REF:2009/63)
8. Comité Etico de Investigación Clínica. (*Ethics in Clinical Investigation Committee*) Hospital Clínico San Carlos, Madrid, Spain. (REF:09/351).
9. Comité Etico de Investigación Clínica. (*Ethics in Clinical Investigation Committee*) Hospital General Universitario Reina Sofía, Murcia, Spain. (REF:14/12/2009)
