# Supplementary material for: Diabetes-specific enteral nutrition formula in hyperglycemic, mechanically ventilated, critically ill patients: a prospective, open-label, blind-randomized, multicenter study
Source: Crit Care. 2015 Nov 9;19:390. doi: 10.1186/s13054-015-1108-1 (PMC4638090; doi:10.1186/s13054-015-1108-1)
Supplement: Additional file 2: Table S1. — Insulin therapy protocol. (DOCX 19 kb) [file 13054_2015_1108_MOESM2_ESM.docx]

**Table S1 Insulin therapy protocol**

**A.- Target**: Capillary glycemia (CG) 110-150 mg/dl.

**B.- Insulin pump:** 50 IU of standard insulin in 50 ml of physiological saline.

**C.- Steps:**

1. Determine baseline CG
2. Start insulin administration according to starting regimen
3. Simultaneously start enteral nutrition
4. Determine CG every hour to adjust infusion rate to starting regimen. After the third hour, change to maintenance regimen.

**D.- Interruption of enteral nutrition**: if for any reason nutrition is stopped, simultaneously close off the insulin pump and continue CG determinations every hour until at least a total of three. Assessment by responsible physician.

**E.- Insulin therapy withheld for > 24 hours**: because CG is in the normal range (110-150 mg/dL); determinations every 6 hours until > 150 mg/dl, from which point therapy is resumed at 1 ml/h following the maintenance regimen. If the target glucose level is maintained after 48 h of withholding therapy, it should be interrupted indefinitely.

**F.- Starting regimen**

| Capillary glycemia (mg/dL) | <150 | 151-200 | 201-250 | 251-300 | >300 |
| --- | --- | --- | --- | --- | --- |
| Infusion rate | - | 1 ml/h | 2 ml/h | 3 ml/h | 4 ml/h |

**G.- Maintenance regimen**

1. Determine CG every 4 h if stable. Switch to every hour following any modification.
2. CG 110-150 mg/dl: do not modify infusion rate.
3. CG >30 mg/dl higher than the previous reading: increase infusion rate by 1 ml/h.
4. CG 1-29 mg/dl higher than the previous reading: increase infusion rate by 0.5 ml/h.
5. CG >30 mg/dl lower than the previous reading: reduce by 1 ml/h only if CG is < 180 mg/dl. Do not modify if CG is > 180 mg/dl.
6. CG 1-29 mg/dl lower than the previous reading: reduce by 0.5 ml/h only if CG is < 180 mg/dl. Do not modify if CG is > 180 mg/dl.
7. CG <110 mg/dl: stop infusion until CG is > 150 mg/dl, restart at 1 ml/h and continue adjustments according to maintenance regimen.
8. CG 50-80 mg/dL: stop infusion, administer 20 ml of glucose 33% and alert responsible physician. If CG is <50 mg/dL, stop infusion, administer 40 ml of glucose 33% and alert responsible physician. Take CG readings every 15 min until equal to or higher than 80 mg/dL.
