# Supplementary material for: Diabetes-specific enteral nutrition formula in hyperglycemic, mechanically ventilated, critically ill patients: a prospective, open-label, blind-randomized, multicenter study
Source: Crit Care. 2015 Nov 9;19:390. doi: 10.1186/s13054-015-1108-1 (PMC4638090; doi:10.1186/s13054-015-1108-1)
Supplement: Additional file 3: Figure S1. — Patient numbers per treatment group during the course of follow-up. (DOCX 12 kb) [file 13054_2015_1108_MOESM3_ESM.docx]

**Figure S1 Patient numbers per treatment group during the course of follow up**
